# The Impact of Food Environments on Midlife Australian Adults With Addictive Eating Behaviors

**DOI:** 10.1177/00332941241303715

**Published:** 2024-11-26

**Authors:** Ruby Eldridge, Rebecca A Collins, Tracy L Burrows

**Affiliations:** School of Health Sciences, 5982 University of Newcastle, University Drive, Callaghan, NSW, Australia; School of Health Sciences, 5982 University of Newcastle, University Drive, Callaghan, NSW, Australia; Hunter Medical Research Institute, Food and Nutrition Research Program, New Lambton Heights, NSW, Australia

**Keywords:** Addictive eating, processed food, fad diets, qualitative

## Abstract

Research into addictive eating has gained traction over recent years, however there is still debate among experts surrounding the nature of the condition. Using reflexive thematic analysis this paper considers addictive eating through a participant focused lens, specifically focussing on the phenomena of the food environment and its impact on individuals with addictive eating. Semi-structured interviews were undertaken to explore the participants’ (*n* = 10) perspectives with addictive eating. After completion of interviews, six main themes were constructed; Relating to the food environment impact was *Convenience of processed foods*, which encompassed two subthemes; *the Easy option,* and, *Essential for survival*; *It’s what I can afford;* Relating to fad diets and addictive eating was *Consciousness; Unmaintainable and unsustainable; Cutting though the noise;* and *Being a part of the group*. This study highlights the need to support consumers to consider ways in which fresh foods can be made more accessible and convenience foods made more healthful, and what support can be provided to food-insecure adults living with food-insecurity.

## Introduction

Addictive eating is a contemporary area of research. However, there is ongoing debate in the literature regarding addictive eating and whether it is more aligned distinctively with behavioural addictions, or substance use disorder or indeed a condition that crosses and moves between these on a spectrum (DiLeone et al., 2012; Gearhardt & Schulte, 2021; Novelle & Diéguez, 2018). To date, addictive eating has been likened to, and mapped to the definitions for substance-use disorders, and behavioural addictive disorders in the Diagnostic Statistical Manual for Mental Disorders (DSM 5) (American Psychiatric Association, 2022) and correlations drawn between disordered eating, specifically binge eating disorder (BED). Individuals who exhibit addictive eating behaviours generally consume lower quality diets with overconsumption of ultra-processed foods, with suggestions that this indicates these foods may have addictive properties and therefore addictive eating be considered as a substance use disorder (Gearhardt & Hebebrand, 2021; Godos et al., 2022; Pursey et al., 2021; Whatnall et al., 2022). Others suggest it is the behavioural act of eating that constitutes a more appropriate definition of addictive eating (Ruddock et al., 2017).

Research to date indicates the current prevalence of addictive eating as defined by the YFAS 2.0 amongst adults is approximately 15% (Oliveira et al., 2021). However, many more adults potentially endorse the recognised symptoms of addictive eating but may not have the clinical impact on daily functioning to meet the criteria. The high prevalence of addictive eating behaviours emphasises the need for continued research in this area.

The food environment is determined by several components, including political, social and physical factors. With the cost of living increasing, lower-socioeconomic communities, and many people in general, are experiencing a lack of access to affordable fresh whole foods, especially those living in rural communities (Foodbank, 2021; Mackenbach et al., 2019; Whelan et al., 2018). This decreased accessibility to fresh foods may result in an increased consumption of foods with a longer shelf life which are less nutritious, and typically classified as processed or ultra-processed (Whelan et al., 2018). These ultra-processed foods have been linked to adverse health outcomes, such as chronic diseases including diabetes, cancer, and cardiovascular disease (Elizabeth et al., 2020; Habibi et al., 2022). Processed and ultra-processed foods can be determined using classification systems such as the NOVA classification system (Habibi et al., 2022; Monteiro et al., 2019) and are highly accessible, resulting in an environment that may promote addictive eating-like behaviours in the general population making it difficult for those susceptible to addictive eating to control food cravings (Monteiro et al., 2013).

Along with the availability of processed and ultra-processed foods, the advertisement and popularity of these foods in addition to fad diets also contributes to the food environment. Fad diets can be defined as trends that promote abnormal eating styles to achieve weight loss, or another health-related goal in a short amount of time (Tahreem et al., 2022). Minimal research has been conducted surrounding the effect of fad diet culture on addictive eating behaviours, however some studies suggest that fad diets may have a role to play in the development of disordered eating behaviours. A review by Stewart et al. (2022) investigating the relationship between dieting, caloric restriction and dietary restraint indicated that these dieting behaviours, comparable to fad diets, may not have strong potential to cause eating disorders in the general population, but following these diets may increase the likelihood of the development of an eating disorder in those who are already at risk. Given the known link between addictive eating and eating disorders such as anorexia nervosa, bulimia nervosa, and BED, it is likely that fad diets, or the diet mindset of quick fixes, may affect those with addictive eating behaviours in a similar way to how these diets affect the development of eating disorders to those at risk (Burrows et al., 2017). This opens the question of whether fad diets, and their influence on the food environment, have an impact on the development of addictive eating behaviours in predisposed individuals.

Presently, research around food choice and addictive eating has been centred in quantitative research, with qualitative research around the impact of the food environment on adults with addictive eating behaviours slowly emerging. As eating is specific to the individual for example, food cravings, reactions to food cues, and impact of fad diet messaging, gaining a richer understanding of the experience of eating in the current food environment is important for future management of addictive eating behaviours (Burrows et al., 2020).

The aim of this study was to investigate the impact that the food environment, with specific interest in the availability of processed foods and the impact fad diet culture have on adults with addictive eating. This exploration of food environments will aid in achieving a better understanding of the experience of addictive eating and management of addictive eating behaviours.

## Methods

### Research design

Reflexive thematic analysis as described by Braun & Clarke (Braun & Clarke, 2006) was used to explore the impact of food environments on the experiences of adults with addictive eating. Reflexive thematic analysis was chosen to contextualise how the phenomena of aspects of the food environment for example, availability of processed foods and exposure to fad dieting information impacts the food and eating choices made by adults with addictive eating.

### Recruitment

Convenience sampling was utilised for the recruitment stage of this research study. A multi prong approach was taken via social media including Facebook and Twitter as well as University notice boards. A post was distributed outlining the aim of the study, and a link to the eligibility questionnaire. Adults who had previously participated in the Targeted Research for Addictive and Compulsive Eating (TRACE) program and had consented to take part in future studies were approached via email (Skinner et al., 2023).

The intention of the study was to recruit ten participants and then determine richness of data once these interviews had been completed. Recruitment ran from March 2023 until June 2023. To be eligible for the study, participants must report three or more symptoms of addictive eating plus clinical impairment as determined by the YFAS 2.0. Other eligibility criteria included being 18 years of age, currently residing in Australia, being proficient in English and have phone/internet access.

### Measures

Participants were first required to complete an online eligibility questionnaire. The eligibility questionnaire was comprised of eight fixed response questions that collected information on the participants’ demographics and the YFAS 2.0 to assess eating behaviours. If participants were deemed eligible, consent to be contacted for the interview was collected at the end of the questionnaire.

#### Demographics

Information collected included the participant’s age; gender (man, woman, other/non-binary or prefer not to say); their level of education (School Certificate, Higher School Certificate, trade or diploma, Undergraduate University Degree, Postgraduate University Degree, Higher Research Degree or no formal qualifications); whether or not they live in Australia; if they are employed (full-time, part-time or casual) or if they are unemployed (looking for work or not looking for work); their relationship status (single, defacto, married, separated, divorced, widowed); whether or not they have dependents; and if they identify as Aboriginal and/or Torres Strait Islander.

#### Eating Behaviors

Following on from these questions, participants completed the YFAS 2.0, a tool developed to assess symptoms and severity of food addiction. The questionnaire is 35 questions to determine food addiction symptoms (0–11). The YFAS 2.0 tool also indicates the severity of the food addiction ranging from no food addiction (1 or fewer symptoms), mild food addiction (2 or 3 symptoms), moderate food addiction (4 or 5 symptoms), or severe food addiction (6 or more symptoms). Before completing the YFAS 2.0, participants were prompted to read through a list of predefined foods (mainly processed foods). Questions include eating habits and how they affect their mental, physical and emotional health and wellbeing; their relationships with friends and family; work, school and other social functions; and how their overeating tendencies affect their daily life in any other ways (Gearhardt et al., 2016; Gearhardt & Hebebrand, 2021).

#### Interviews

Interviews were undertaken to gain an understanding of the participants’ experience with addictive eating and how they felt it was affected by the food environment. The interview questions were developed by RC, who has been working in the field of addictive eating for six years and has previously undertaken qualitative research in addictive eating behaviours. RE was a student dietitian who received guidance and training from RC and the research team. The interviews were conducted by RE and RC. Neither RE or RC had a prior relationship with the participants. The extent of the participants’ knowledge of the interviewers was that they were dietitians completing a research study. A semi-structured interview guide (Table 1) was developed with the interviewer providing additional prompts and probes for the participant to develop on their response to the structured questions. The participants were provided with the interview guide before their interview took place to familiarise themselves with the questions. Informed consent was obtained prior to recording of the interviews. The interviews took approximately 30 minutes and were conducted over Zoom or as a regular phone call, depending on the preference of the individual participant. The interviews were audio-recorded, and then transcribed using the Outscribe transcription service (Outscribe https://www.outscribetranscription.com.au). Transcripts were manually checked by RE for accuracy. Identifying or potentially identifying information was removed from transcripts to protect participants’ anonymity. Only the interviewer and the participant were present for the interviews. Field notes were made, where appropriate, throughout the interview process.

**Table 1.** Semi-Structured Interview Guide.

Semi-structured interview guide
1. When I use the phrase “highly-processed foods”, what foods do you think of?
2. How has increased availability of highly processed foods impacted on your eating?
3. How do you think your eating would be different if highly processed foods weren’t as available?
4. When I use the phrase “fad diets”, what comes to mind?
5. What impact have fad diets had on your eating?
6. Is there anything else you would like to add about highly processed foods or fad diets and their impact on your eating?

This study was approved by the University of Newcastle Human Research Ethics Committee (H-2023-0021).

The interview questions were initially developed by RC and then reviewed by the research team. The interview questions were not modified at any stage throughout the interview process.

### Data analysis

RE and RC analysed the data using NVivo 12 qualitative analysis system. The process outlined by Braun & Clarke (2006) for reflexive thematic analysis was followed (22). The steps of this process included:

(1) Familiarising self with data: RE and RC read and re-read the interview transcripts and took note of any pieces of information that initially stood out. They then met to discuss the first read-through.
(2) Generating initial codes: RE and RC separately coded transcripts using NVivo qualitative analysis software (Lumivero, 2023). Each line of the transcript was examined, and then the data was assigned codes to describe it. Initial codes were then grouped together based on similarities to organise them into categories. RE and RC met again to compare codes and discuss the findings.
(3) Searching for themes: RE and RC individually constructed prominent themes and began to describe how the data fell into these themes.
(4) Reviewing themes: RE and RC met again to compare themes with cross-checking completed to ensure the themes were actually present in the participant transcripts. This process was used for RC to support RE as a student researcher.
(5) Defining and naming themes: Themes were then grouped and named, and sub-themes were identified. RE and RC discussed themes once more, before presenting them to the rest of the research team to ensure all data was represented, and themes were clear and accurate.

### Reflexivity

Participant transcripts were analysed by RE and RC. Transcripts were initially read and re-read through by RE and RC to gain a broad understanding of the content. RC has 6 years of experience working with adults with addictive eating and has previously undertaken qualitative studies researching adults with addictive eating behaviours. RC provided ample guidance for RE as a student researcher. Both RE and RC kept reflexive journals throughout the coding process and were in regular contact with each other to ensure their findings were representative of the interview data.

## Results

### Demographics

The age of participants recruited ranged from 40-63 years. The mean age was 50 years, (SD = ±8.1). Seven females and three males participated in this study. The YFAS 2.0 symptom score ranged from 3-11 among the ten participants. The mean YFAS 2.0 symptom score was 8 (SD ± 2.8).

Thirty-seven participants who completed the survey were eligible to undertake an interview. Of this 37, 23 participants consented to being contacted for an interview. All 23 consenting participants were contacted with ten participants booking an interview time. From the ten interviews the six themes constructed are outlined in Table 2. Questions pertaining to fad diets and the availability and accessibility of processed foods did not appear to be interrelated, so were treated as independent aspects of the food environment for analysis.

**Table 2.** Themes From Participant Interviews on the Food Environment.

Themes
Food environment impact
1. Convenience of processed foods
• the easy option
• essential for survival
2. “It’s what I can afford”
Fad diets
3. Consciousness
4. Unmaintainable and unsustainable
5. Cutting through the noise
6. Being a part of the group

Processed foods:

(1) *Convenience of processed foods* was a popular concept raised during the interviews when participants were asked about the impact of highly processed foods on their eating. Convenience was commonly described to be a benefit of processed foods, with many participants reporting that the high level of convenience associated with highly processed foods makes their lives easier. In some cases, this convenience was even described to be essential for survival. However, some participants described this convenience as a negative as they now rely on the convenience of processed foods.

### The easy option

Many of the participants described the ease of access to processed foods and how particular aspects of their live were made easier by having access to processed foods.

Participant 53 stated, when asked about processed foods:

“The main reason I eat it is because it’s easy and it’s there and you can have it in your room.”

Participant 67 had a similar response and explained how choosing processed foods was related to their mental health and how having access to processed foods and the minimal preparation steps involved is beneficial to them:

“I think it’s easy to grab and have a stash of food in the cupboard, last minute food… I live with some mental health issues and so managing food, meal planning is challenging. Those foods that are highly processed are easier options.”

Participant 95 referred to the difficult choice between choosing an unprocessed, fresh food which would usually be a healthier option, over a highly processed, likely less healthy food which is more convenient but arguably more palatable:

“I give myself the choices to have both processed or unprocessed food, and because the others are more appealing, like eating the biscuits or the ice cream or whatever is a more appealing thing than a pear or a... It tends to be the one that people reach for if it's in a home because it's more convenient and it's more delicious.”

Participant 53 reflected on what it might be like if the easy option of processed food was not as available:

“If you wanted to eat you’d have to get it ready which is what happens. I can’t afford to get, if I’d run out of that stuff or whatever then I’d just have to go out in the kitchen and make something.”

### Essential for survival

Participant 38 referred to their marriage breakdown and how it made food preparation a struggle. The convenience of processed foods was essential for this individual to function and support their children. They explained:

“My ability to function, my ability to be part of everyday society in a normal way, and a lot of the times because of financial restraints or restrictions, I was looking for things that I could access for my family quickly, because I was in no frame of mind to be spending long amounts of time cooking, thinking, preparing, it wasn’t enjoyable for me.”

Participant 67 discussed this from the perspective of eating versus not eating:

“…but there’s people out there who they actually, if it was between not eating and eating a processed food then at least you’re eating.”

The same participant also expressed how a lack of processed foods would impact their life:

“I imagine that if the only options were fresh food…I feel like I would need to spend more of my time preparing and planning meals, and even shopping, I would go to the shops more often… I don’t even have a family, but I just feel like oh my gosh, that would feel like so much pressure… I feel like I’d have to work less”

These participant experiences show that processed foods are easy food options that from a behavioural perspective have both pros and cons. Individuals are often considering the interplay between feeding themselves or going without as food preparation is an ongoing lifestyle barrier.

(2) *“It’s what I can afford”* was a common sentiment raised in the interviews when participants were speaking about highly processed foods. The economic climate has seen a significant rise in the cost-of-living in recent years (Broadbent et al., 2023). This results in changing food environments and the transition of fresh foods, which would be traditionally considered ‘healthier’ options become perceived as more expensive, and less healthy, processed foods become the perceived cheaper options. (Foodbank, 2021)

Participant 67 described the cost benefits of processed foods in relation to their non-perishable nature:

“In some ways I think that the thought of processed food, they tend to feel more affordable and available and don’t waste as much.”

Feeding an entire family can cause high levels of financial stress. Fussy eating or simply children’s preferences play a role in this as well.

Participant 38 described their experience:

“It was just grab what you could get for the money I could get, and I knew that the kids were going to need for school or for snacks or that they ate, things that they would… That's not just fast foods but absolutely things packaged ready to go in the supermarkets, and a lot of the time not just convenience price point, what I could manage to afford.”

This notion of pre-packaged foods being cheaper is something that has only emerged in recent years. With the increasing cost of living, cheap fast food, which is generally considered to be processed, has become more appealing to families. The current food environment is characterised by the high availability and accessibility of well-known fast-food chains leading to higher consumption levels across the population. Participant 33 described their situation:

“…we don’t have a great deal of spending money. We own a house, oh well we’re paying for mortgage so, and that’s high at the moment and everything else is high, and so we, if we do need some food and it’s in a hurry it’ll be one of those kind of food places that are cheaper.”

Participant 95 compared the price of fresh foods with the price of processed foods:

“…households that have to be careful of what they spend find that the processed foods are cheaper to manage than the unprocessed, real, fresh foods are becoming more and more expensive.”

Participant 97 spoke about the price of fruit and vegetables in Australia compared to where they were previously in the German countryside:

“Then when I moved back to Australia, and that was in 2015, I found it really hard to access fresh fruit and vegetable at a price that I could afford.”

Participant 97 then described how easy it is to find affordable, processed foods, and how this is not the case for healthier alternatives:

“I have to make a choice between having something closely healthy and affordable and I can find it, or I have to go out of my way to find something that I can afford that is not processed or preserved or it comes as a preprepared meal.”

They continued to speak about their experience with the availability of processed foods once they had moved back to Australia:

“I didn’t have the money to go and get a taxi to go to the place where it would be more affordable like the markets, but at the same time also I just didn’t have the money to go to XX and buy fresh fruit and vegetables, or fresh food, it was more affordable for me to get something that is already prepared and processed, even though it’s, if I had cooked it myself it would probably be healthy.”

Later in the interview, participant 97 spoke more about the impossibility of accessing affordable fresh foods:

“You’ve got to find it now, like you’ve got the obvious spots, but you’ve really got to find it to be healthy and affordable and not, if you’ve got a budget and you’re trying to eat, like full stop.”

Most of the participants discussed fad diets as something they knew about and had experimented with, but were not overly invested in. Some participants had undertaken fad diets in the past, with a combination of positive and negative outcomes from undertaking these types of diets. For some participants it assisted them to know how to eat better overall, however, others have experienced a loss of understanding around what a good relationship with food looks like.

(3) *Consciousness -* Som*e* participants felt that fad diets made them more conscious of what they were eating and how they were fuelling their bodies.

Participant 92 spoke on this:

“It’s teaching you to value what you’re eating. So, I mean I think whatever ends up happening it’s you’re changing your perception of what eating is about. So I think it makes you realise how important your diet is. It doesn’t help me have the discipline around changing what I eat but it changes my consciousness. I’m thinking more about what I’m eating rather than just taking it for granted.”

And participant 33 had a similar response. They reflected on their experiencing with dieting:

“…yeah more conscious of what I was putting in myself.”

(4) *Unmaintainable and Unsustainable -* A common response was that fad diets are not suitable in terms of eating or for the long-term weight loss they expressed to want. Many participants felt that these diets do not produce any lasting health benefits, and that they are unmaintainable and misinformed. Fad diets seem to create a ‘cycle of dieting’, where a diet is successful for a short period of time, but then this success begins to plateau. This often causes the individual partaking in the fad diet to stop the diet and resume their original eating behaviours. The individual will then feel the need to try something new and the cycle continues.

Participant 53 described the unmaintainable nature of fad diets, and how this can result in a cycle of dieting:

“Sort of like things that make you try and lose, things that you can lose weight but if you don’t maintain it as soon as you stop doing it all the fat comes back again…Sometimes you see it and you give it a go and you might lose a little bit to start with and then it just plateaus or you can’t maintain it. So, they're not maintainable”

When asked about their experience trying a fad diet, Participant 67 stated:

“It did have some success for me, but again, I think the downside of those types of programs is sustainability. It doesn’t really change eating behaviours.”

Participant 59 explained how following fad diets was not maintainable for them with their lifestyle:

“…in the long term with children and, you know 2 of my children have special needs, time wise and track and that just, you know, yes it was effective while I was doing it but sticking onto it long term just wasn’t, didn’t fit in with my household and my children really.”

Participant 92 had a negative outlook on fad diets and their unmaintainable nature:

“And so, I think your question what is a fad diet. It’s an unhealthy…you could say media driven frenzy around something that may be beneficial but there’s no real long term benefits because people can’t maintain the fad.”

This participant then went on to say:

“And although those, I can see the no sugar and the, which I’ve done 3, probably 3 odd times… it does have a great benefit… but I can’t maintain it long term.”

Participant 95 had this to say about the sustainability of fad diets, specifically meal replacement diets:

“It was exciting to actually feel physically better. And I think they also have an appetite suppressant in them so that you don't miss finding others, you don't go looking for anything. I'm not sure even if it's because your mind’s in the zone or because there's the appetite suppressant, but you're just not hungry on them. It's just crazy. But then it's unsustainable. You can do that for three or four months and then you start going, oh, I'll have to do that.”

They then went on to speculate about the reason for this unsustainability:

“And I don't know why they're not sustainable because I think there's something I've read, because I do read a bit about this kind of stuff, that there's something where your hormones change at about the three-to-four-month mark to say, we need something else going on here. This diet's been the same for four months, let's do something else.”

(5) *Cutting through the noise* - A common sentiment among adults with addictive eating behaviours is that fad diets spread nutrition misinformation. It can become difficult to decipher the sound nutrition advice from the misinformation, which can make developing healthy eating behaviours confusing for many individuals.

Participant 33 spoke about this difficulty to know where the truth is, and how everyone sharing their differing opinions can make things confusing:

“Yeah some people say like have small, 6 small meals a day, like you might have a boiled egg here and a bit of cheese there and a bit of fruit there and then other people are like no you need to leave like time for your body to digest in between 3 meals a day and, and this is from people that, you know, that have like worked in gyms that I’ve asked some advice for like trainers and everyone’s got a different opinion and it’s so frustrating.”

Participant 96 displayed their mistrust for fad diets as they often come from unqualified people:

“I suspect I'm a little unusual in that I have a disrespect for the term and for the diets and I suspect a lot of people would think that they're genuine and something to try, but to me it's just a disreputable term from people who don't know what they're talking about. So, I wouldn't go near them. If I wanted dietary advice, I'd see a healthcare professional.”

(6) *Being a part of the group* was a theme that popped up when participants were questioned regarding their experience with fad diets. Participants linked that society accepts a certain body type as the beauty standard, and those who do not fit into this category felt the need to step into the fad diet culture to try and fit in.

Participant 38 spoke of their struggle, coming from a Mediterranean background, with trying to fit this ideal Australian body type and how they turned to fad diets with the hope of coming to fit this beauty standard:

“Growing up I came, like I said, from a Mediterranean background. My identification with what ideal bodies in Australia looked like I didn’t fit that mould, so I was constantly drawn to lose so many kilos in one week or follow this diet for a month and you’ll lose so much stones.”

Another example of this notion of ‘fitting in’ in the context of fad diets, is following others around you in their dieting pursuits. Participant 53 spoke of their experience with fad diets, specifically meal replacement shakes, when they were regularly going to the gym and surrounding themselves with people who had similar fitness goals:

“In that stage of my life I was going to the gym and that so it was a fit sort of thing, so all the gym guys were doing it, so you felt part of the group and because it was working and that it made you feel a bit better.”

## Discussion

This study set out to gain an understanding of how the food environment, more specifically the availability and accessibility of processed foods, and the culture surrounding fad diets, has impacted on adults in Australia with addictive eating.

Following semi-structured interviews, six main themes were extracted from the analysis; convenience of processed foods, “it’s what I can afford”, consciousness, cutting through the noise, and being a part of the group.

Results of this study found common experiences among adults with addictive eating in the current study to struggle with the choice between what is healthy, and what is affordable, both to feed themselves and to feed their families. This result was identified in the current population group of adults with addictive eating however might also be reflective of adults in general, rather than specifically to those with addictive eating. However, as adults with addictive eating have been shown to consume higher amounts of processed foods and lower diet quality and rates of addictive eating are more prevalent in food insecure populations, this difficulty in making the choice between healthy and affordable may be exacerbated in addictive eating populations (Gearhardt & Hebebrand, 2021; Godos et al., 2022; Parnarouskis et al., 2022; Pursey et al., 2021; Whatnall et al., 2022). For example, in the current economic climate, the cost of living in Australia, and in many places internationally, in 2023 has increased dramatically, and is continuing to rise (Foodbank, 2021). Many participants reported that processed foods are often their first choice for convenience, as they are much more financially accessible than fresh foods. This encompasses fast food, which was reported to be more appealing than making meals at home from scratch due to its cheap and convenient nature. A study undertaken in 2016 by Okrent and Kumcu found that the lower cost of processed foods was the main driver of the high consumption of these particular foods in the United States (U.S) (Okrent AM, 2016). The study found that ready-made, highly processed convenience meals were more appealing to families as they were perceived cheaper than buying each ingredient that would be required to make the meal from scratch. It was also reported that in the U.S, the average price indexation of ingredients was increasing at a more rapid rate than the price of convenience foods, which is similar to the findings of the Australian Foodbank Hunger report (Foodbank, 2021; Okrent AM, 2016).

Furthermore, as cost was identified to have a large role to play in the food choices in the current sample of adults with addictive eating, it is important to consider the socioeconomic status of the participants involved in this study. When considering the demographics of individuals included in this study, they were mainly of a higher education status, and the majority of participants were employed. Therefore, it can be assumed these individuals’ views and perspectives may not be reflective of those from lower socio-economic statuses. However, results may be more pronounced in individuals from lower socio-economic classes, as food addiction behaviours have previously been reported to be higher in those with low income (Parnarouskis et al., 2022). For example, a study by Parnarouskis et al., published in 2022 found that food insecure individuals were more likely to exhibit addictive eating symptoms than non-food insecure individual (Parnarouskis et al., 2022). Their study found that pregnant women who were suffering with food insecurity had a 21% higher addictive eating symptom score than their counterparts who were living in food secure households. Findings also displayed a 56% higher addictive eating symptom score among food-insecure women who are primary caregivers than their food-secure counterparts (Parnarouskis et al., 2022).

Another aspect of processed foods identified in the current study that adds to their level of convenience and cost-effectiveness is their unlikeliness to contribute to high levels of food waste. This was a common concept brought up by participants in the interviews when questioned about processed foods and how they contribute to their eating behaviours. Research has shown that environmental, moral, financial and social concerns all contribute to an individual’s desire to avoid food waste (Ribbers et al., 2023). Participants of this study linked the non-perishable nature of processed foods with their cost-effectiveness, indicating that their motivations to choose foods which are less likely to contribute to food waste are mainly financial. This may have particular relevance to the addictive eating population due to their increased likelihood of experiencing food insecurity (Parnarouskis et al., 2022).

The majority of people in the current study identified as women which may have also contributed to findings around the cost of food and convenience being a driving factor of consumption of highly processed foods. Women have increasing roles in the workforce, and mothers are met with heightened challenges regarding providing for their families (Reich-Stiebert et al., 2023). Women may look increasingly to convenience foods as they are the time saving option in a society that expects mothers to provide healthy options for their kids but does not provide the support for mothers to achieve this (Reich-Stiebert et al., 2023). The choice to buy highly processed, convenience foods appeared to be one which was commonly referred to as a need for survival by the women in the current study. With female caregivers having a 56% higher likelihood of displaying symptoms of addictive eating (Parnarouskis et al., 2022). The increased intake of processed convenience foods as a method for survival may result in a perpetuation of addictive eating behaviours in individuals in these circumstances. Alternatively, these women may be displaying addictive eating symptoms already and choose highly processed convenience foods primarily because these are the foods they are craving, and consequently perceive these foods to be essential for survival. This is an area which would benefit from further research.

A common concept that arose during the interviews with respect to fad diets was that they make an individual more conscious of the foods they are putting in their body. This increased consciousness or awareness of what one consumes may be considered to be beneficial to certain members of the populations’ health outcomes, and a good starting point often associated with the contemplative stage of the behaviour change model (Queensland Government, 2007). However, for others, this increased consciousness of the foods they are putting into their body can create an obsession/drive towards food which could contribute to poor eating behaviours which is highly relevant considering a constant desire for food alongside craving are symptoms associated with addictive eating (Penzenstadler et al., 2019).

Participants related fad diets to the notion of fitting in and the desire to feel as though they were a part of a group they desired to belong to or a ‘quick fix’ for those with a higher weight status, as a relief from feeling different or left out. Participants in this study spoke of their experiences coming from diverse cultural backgrounds and moving to Australia where there was a beauty standard that seemed unattainable for a lot of people. Fad diets, with their promises of week-by-week weight loss, have a level of desirability for people who do not fit into self-perceived social beauty standards. A study on female models found that pressures to fit societal beauty standards were a driver for these individuals to follow fad diet trends (Vidianinggar et al., 2021). This can be related to the findings of this study, as it can be hypothesised that this similar pressure is felt by the participants who stated they did not fit in with the ideal body type and turned to fad diets to change this.

### Practical Implications and Recommendations for Future Research

This study found that processed foods have a significant impact on the participant’s eating behaviours, due primarily to their highly convenient nature. In most cases, it appears that processed foods make life easier for many people, and in some cases have even been associated with survival and contributed to less waste. Further research is needed to determine how healthier alternatives can be made just as convenient as processed foods and therefore equally as appealing in that regard. It also suggests that these are barriers that individuals face when attempting to change their behaviours to more healthier choices. Ideally, this may result in improved health outcomes for those living with addictive eating.

The current study shows cost as a prominent reason which adults living with addictive eating appear to choose processed foods over fresh foods. At the current time of writing, Australia is in a cost-of-living crisis (Broadbent et al., 2023). Future research surrounding whether processed food intake has increased further among the addictive eating population since this rapid increase in the cost of living would be recommended.

### Strengths and Limitations

There were notable limitations of this study including small sample size, and higher numbers of women. Only 10 participants were included in the study, however this is consistent with other qualitative studies. This means that results are not generalisable to the broader population. None of the participants identified as Aboriginal and/or Torres Strait Islander, which means this study does not represent the views of food environments on addictive eating within the First Nations population. All participants in this study identified as either male or female. The lack of non-binary participants also made the results less generalisable to the entire population of the target group. Additionally, the age demographic of participants in this study did not encompass anyone born after 1983. As fad diets are ever-evolving trends, individuals who grew up in more recent times may have a different perspective on fad diets than what was conveyed in the results of the current study. The current study allowed participants to have their own definitions of what they viewed as processed food and fad diets meaning the definition of these terms could be varied for each participant.

## Conclusion

The current study identified that addictive eating continues to be an area requiring further investigation and research. The availability and accessibility of highly processed foods and fad diet culture appear to have both negative and positive impacts on adults with addictive eating and their eating behaviours. No links were found between the two focus areas of the food environment of this study; fad diet culture and the availability of ultra-processed foods, however both components appeared to impact on the participants’ experience of eating in a general sense. Further research should be done to investigate how other aspects of the food environment impact on eating behaviours among the addictive eating population.

## Data Availability

The datasets generated during and analyzed during the current study are available from the corresponding author on reasonable request.*
